# Model of end stage liver disease (MELD) score greater than 23 predicts length of stay in the ICU but not mortality in liver transplant recipients

**DOI:** 10.1186/cc9068

**Published:** 2010-06-15

**Authors:** Christian E Oberkofler, Philipp Dutkowski, Reto Stocker, Reto A Schuepbach, John F Stover, Pierre-Alain Clavien, Markus Béchir

**Affiliations:** 1 Department of Visceral- and Transplantation Surgery, University Hospital of Zurich, Raemistrasse 100, Zürich 8091, Switzerland; 2 Surgical Intensive Care Unit, University Hospital of Zurich, Raemistrasse 100, Zürich 8091, Switzerland

## Abstract

**Introduction:**

The impact of model of end stage liver disease (MELD) score on postoperative morbidity and mortality is still elusive, especially for high MELD. There are reports of poorer patient outcome in transplant candidates with high MELD score, others though report no influence of MELD score on outcome and survival.

**Methods:**

We retrospectively analyzed data of 144 consecutive liver transplant recipients over a 72-month period in our transplant unit, from January 2003 until December 2008 and performed uni- and multivariate analysis for morbidity and mortality, in particular to define the influence of MELD to these parameters.

**Results:**

This study identified MELD score greater than 23 as an independent risk factor of morbidity represented by intensive care unit (ICU) stay longer than 10 days (odds ratio 7.0) but in contrast had no negative impact on mortality. Furthermore, we identified transfusion of more than 7 units of red blood cells as independent risk factor for mortality (hazard ratio 7.6) and for prolonged ICU stay (odds ratio [OR] 7.8) together with transfusion of more than 10 units of fresh frozen plasma (OR 11.6). Postoperative renal failure is a strong predictor of morbidity (OR 7.9) and postoperative renal replacement therapy was highly associated with increased mortality (hazard ratio 6.8), as was hepato renal syndrome prior to transplantation (hazard ratio 13.2).

**Conclusions:**

This study identified MELD score greater than 23 as an independent risk factor of morbidity represented by ICU stay longer than 10 days but in contrast had no negative impact on mortality. This finding supports the transplantation of patients with high MELD score but only with knowledge of increased morbidity.

## Introduction

Liver transplantation is still a complex and cost-intensive procedure [1] and the results are influenced by many interrelated factors. As liver transplantation has become a universally accepted treatment for end-stage liver disease, the number of patients accumulating on the waiting list has gradually outweighed the scarce resources of available organs. Fair allocation of donor livers to patients with end-stage liver disease is a difficult task. The USA and Europe used prioritization systems based on waiting time and on the parameters of the Child-Turcotte-Pugh score [2]. Since February 2002, the United Network for Organ Sharing introduced a new allocation policy for cadaveric liver transplants, based on the model for end-stage liver disease (MELD) score [3]. This new policy stratifies the patients based on their risk of death while on the waiting list [4]. The impact of MELD score on postoperative mortality remains elusive. There are reports of reduced survival in groups with high MELD scores [5,6], but also reports of no influence of MELD score on survival [7,8].

Furthermore, the unique pathophysiology of end-stage liver disease (ESLD) has important implications on critical care treatment after transplantation [9]. Although liver transplantation has been the sole treatment of patients with ESLD for over 20 years, only limited data are available addressing the intensive care management and complications of this patient population [10,11].

The current challenge is to optimize outcome with limited resources, because liver transplantation remains financially expensive with incremental costs when postoperative complications occur. Therefore, it is essential to identify and modify risk factors to improve postoperative ICU management.

In this study we addressed the question of whether MELD score affects postoperative morbidity, represented by an increased length of stay in the ICU and mortality in patients after liver transplantation. Furthermore, the study was undertaken to determine the major ICU problems in such patients and to outline and predict major clinical risk factors regarding length of stay in the ICU and mortality.

Therefore, data from all consecutive liver transplants performed in our institution over six years, from 1 January 2003 to 31 December 2008, were analyzed.

## Materials and methods

We included in the study a total of 144 consecutive patients who underwent liver transplantation between 1 January, 2003 and 31 December, 2008 in our transplant center. Five of these patients underwent seven retransplantations. Two of them underwent retransplantation twice and three patients only once, and two cases out of this seven were electively listed and five patients were high urgent listed. Thus, we included data of 151 liver transplantations in 144 patients over six years with a median follow up of 27.0 months into our study.

Patients were transplanted according to the MELD score, which is based on recipient kidney function, coagulation time and serum bilirubin, and ranges from 7 to 40. This score is a reliable parameter to predict mortality of liver transplant candidates on the waiting list [12]. In order to prevent discrimination of patients on the waiting list with a hepatic tumor or a metabolic and cholestatic disease, those patients received exceptional points, resulting in higher (corrected) MELD scores than the calculated laboratory (uncorrected) MELD would be [13]. Following approval by the local ethics committee, all patients gave written informed consent before transplantation for postoperative data analysis.

### Inclusion/exclusion criteria

We included all adult (> 16 years of age) liver transplant recipients from January 2003 until December 2008 who were electively or high urgently listed. The only exclusion criteria were living related liver transplant recipients. One patient, who was retransplanted twice (electively listed) during this period was excluded from analysis, because the initial transplantation was before the study period.

### Pretransplant recipient data

We defined extended donor criteria (marginal grafts) as either age 65 years or older or cold ischemia time of 720 minutes or longer or biopsy-proven steatosis (micro- or macrovascular in ≥60% of hepatocytes or ≥30% macrovascular steatosis) [14,15].

As baseline characteristics we analyzed age, gender, height, weight, body mass index, creatinine, hematocrit and platelet count. Creatinine values of the patients with renal replacement therapy (RRT) prior to transplantation were excluded from the calculation. For analysis the last available values directly before transplantation were included. Furthermore, the following clinical data were collected: underlying liver disease, Child-Turcotte-Pugh classification, MELD score uncorrected and corrected for hepatocellular carcinoma according to the regulation of the government [13], incidence of hepatorenal syndrome directly before transplantation (according to the definition described by Arroyo and colleagues [16] and Salerno and colleagues [17]), and diabetes mellitus, electively or high urgent listing, pretransplant location (home, normal hospital ward or ICU) and finally the need for pretransplant RRT.

### Operative data

All patients were transplanted without veno-venous bypass, as described by McCormack and colleagues [18]. Management of coagulation and transfusion practice was performed according to the internal guidelines. Patient data were collected in respect to operating time, estimated intraoperative blood loss, transfusion of red blood cells (RBC), fresh frozen plasma (FFP) or platelets and intraoperative application of fibrinogen.

### ICU data

The following data were collected: length of stay in the ICU, incidence of readmission to the ICU, readmission cause, serum creatinine peak level, incidence of renal failure assessed by the RIFLE (risk, injury, failure, loss, end-stage of kidney disease) criteria, incidence of RRT, incidence of sepsis, incidence of pulmonary failure (acute respiratory distress syndrome (ARDS), pneumonia with consecutive reintubations), ventilation days, serum peak values of bilirubin, alkaline phosphatase, alanine aminotransferase (ALT) and aspartate aminotransferase (AST); incidence of primary graft nonfunction and retransplantation, incidence of rejection on the ICU and reoperations during the ICU stay, and the incidence of acute coronary syndrome. In the case of four primary graft nonfunctions in the ICU with a following four consecutive emergency retransplantations, we considered those four retransplantations as ICU complications and analyzed these patients as four ICU cases. Furthermore, we considered three electively listed retransplantations as three additional cases and therefore calculated the ICU parameters from 147 transplantation cases out of 144 patients. The graft specific parameters, that is peaks of bilirubin, alkaline phosphatase, ALT and AST, were analyzed from all 151 transplanted grafts.

### Analysing protocol

#### Influence of MELD

The influence of patients MELD score on postoperative mortality and length of stay in the ICU longer than 10 days (morbidity) was univariately and multivariately analyzed in 128 electively listed and transplanted patients. High urgent listed patients were not included in these analysis because of another allocation system according to the Clichy criteria [19].

#### Graft survival, mortality

We analyzed data in respect to graft survival after one year, three years and five years and patient's survival was calculated for one year, three years and five years, respectively. Furthermore, the ICU and hospital mortalities (mortality during the hospital period of the transplantation in our center without transfers to other hospitals) were analyzed. For graft survival we analysed the data of all 151 transplantations and all the 144 patients were included in the survival analysis.

#### Identifying risk factors

We performed a Cox proportional hazard model to identify risk factors for mortality of liver transplant recipients. Through multiple logistic regression analysis we identified predictive factors for ICU length of stay of more than 10 days.

### Statistical analysis

MELD influence on mortality and length of stay in the ICU of more than 10 days was univariately performed with an unpaired t-test. For multivariate analysis we used the method of multiple logistic regression to identify risk factors for length of stay in the ICU and a Cox proportional hazard model to identify independent risk factors for mortality. Calculation of mortality and graft survival was performed by Kaplan Meier analysis. We calculated the baseline characteristics, operative parameters, incidence of ICU complications, rejections and reoperation incidence as the relative and absolute numbers. Data are expressed as mean ± standard deviation; different data expression is stated in the text. All calculations were performed with Statview 4.5 (abacus concepts, Berkeley, CA, USA). Statistical significance was accepted with *P *< 0.05 (two-sided tests).

## Results

### How were the pretransplant baseline conditions?

The baseline characteristics of the recipients are shown in Table 1. The underlying liver diseases of the 144 patients are presented in Table 2. The incidence of hepatorenal syndrome and diabetes mellitus was 29 patients (20.1%) and 26 patients (18.1%), respectively. The mean MELD score of these 128 patients was corrected 19.5 ± 7.1 (median 19, range 8 to 40) and uncorrected 15.8 ± 8.6 (median 15, range 6 to 40), respectively. Sixteen out of 144 patients (11.1%) or 21 out of 151 transplantations (13.9%) (inclusive of four retransplantations) were high urgent listed and transplanted because of acute liver failure or primary graft nonfunction, respectively. The location of the patients directly before transplantation was 106 (70.2%) at home, 18 (11.9%) on a normal ward and 27 (17.9%) on the ICU. The incidence of pretransplant RRT was 7 out of 144 patients (4.8%).

**Table 1:** Baseline characteristics (n = 144 patients)

Men	110 (76.4%)
Women	34 (23.6%)
Weight (kg)	77.5 ± 16.1 (43-136)
Height (m)	1.73 ± 0.10 (1.50-1.95)
BMI (kg/m^2^)	25.8 ± 4.3 (16.0-42.9)

Creatinine (μmol/l)	102 ± 56 (40-509)
Hematocrit (%)	32.4 ± 6.6 (15.3-49.6)
Platelets (10^3^/μl)	104 ± 60 (22-285)

Data expressed as mean ± standard deviation (range). BMI, body mass index.

**Table 2:** Underlying liver diseases (n = 144 patients)

HCV liver cirrhoses overall	54 (37.5%)
HCV liver cirrhoses + HCC	20 (13.9%)

HBV liver cirrhoses overall	16 (11.1%)

HBV liver cirrhoses +HCC	7 (4.9%)

HCC overall	41 (28.5)

Alcoholic liver cirrhosis overall	24 (16.7%)

Alcoholic liver cirrhosis + HCC	1 (0.7%)

Alcoholic liver cirrhosis + HBV	1 (0.7%)

Acute liver failure	12 (8.3%)

PSC	5 (3.5%)

PBC	4 (2.8%)

Morbus Wilson	4 (2.8%)

Cryptogenic liver cirrhosis	2 (1.4%)

Amyloidosis	3 (2.1%)

Budd chiari syndrome	2 (1.4%)

Alpha-1-antitrypsin deficiency	1 (0.7%)

AIH liver cirrhosis	1 (0.7%)

Polycyclic liver disease	1 (0.7%)

Hyperoxalurie	1 (0.7%)

Vanishing bile duct syndrome	1 (0.7%)

M. Osler	1 (0.7%)

AIH, autoimmune hepatitis; HBV, hepatitis B virus; HCC, hepatocellular carcinoma; HCV, hepatitis C virus; PBC, primary biliary cirrhosis; PSC, primary sclerosing cholangitis.

The mean age of donors was 48.6 ± 17.1 years and the cold ischemia time was 539 ± 166 minutes. According to the chosen criteria for extended donor grafts 57 out of 151 (37.7%) marginal donor grafts used in our study population showed at least one of the defining criteria.

### How was the intraoperative management?

The mean operation time for the 151 transplantations was 391 ± 90 minutes (median 370, range 280 to 705). The estimated blood loss during the operating procedure was 2,559 ± 2,860 ml (median 1,300, range 200 to 15,000). Transfusion requirements during transplantation were 6.2 ± 8.1 units of RBC (median 4, range 0 to 47), 14.2 ± 12.9 units of FFP (median 12, range 0 to 77), 1.7 ± 2.9 units of platelets (median 1, range 0 to 18) and fibrinogen 3.2 ± 5.1 g (median 0, range 0 to 22).

In a total of 117 (81.8%) transplantations RBC were transfused, in 133 (86.9%) FFP and in 71 (50.7%) platelets were given. No transfusion of RBC, FFP or platelets was achieved only in seven (4.6%) transplantations. Fibrinogen was administered in 76 (49.6%) transplantations.

### Did MELD affect postoperative course?

The analysis of the 147 ICU cases showed a mean initial ICU length of stay of 8.8 ± 13.6 days (median 4, range 2 to 94), a readmission rate of 34 (22.8%), whereas 7 patients were readmitted twice and one patient 4 times. The mean readmission length of stay was 2.0 ± 6.5 days (median 0, range 0 to 50) and in turn the overall length of stay in the ICU was 11.3 ± 16.1 days (median 5, range 2 to 96). The serum creatinine peak level in the ICU was 174 ± 91 μmol/l (median 155, range 64 to 429). The incidence of renal failure according to the RIFLE criteria in the 137 ICU cases without pretransplant RRT was: class 1 (risk) 26 (19.0%), class 2 (injury) 26 (19.0%), class 3 (failure) 34 (24.8%) and class 4 (loss) 9 (6.6%), with overall 95 (69.3%) patients presented with renal failure in different stages.

**Figure 1:**
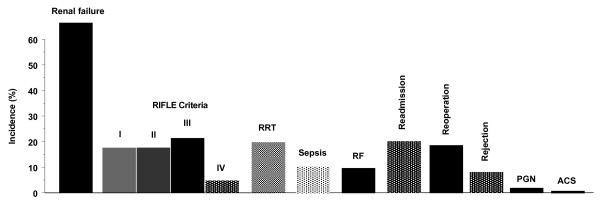
**ICU complications of the 147 ICU cases**. ACS, acute coronary syndrome; PGN, primary graft nonfunction; RF, respiratory failure; RRT, renal replacement therapy.

RRT was necessary in 32 (21.8%) of the transplanted patients at the initial ICU stay and in 33 patients (22.4%) over all ICU days together, inclusive of readmission time. Ventilation days during the ICU stay were 4.7 ± 10.5 days (median 2, range 1 to 80). The ICU complications were: sepsis in 16 patients (10.8%), respiratory failure (ARDS, pneumonia, reintubation) in 15 patients (10.2%), primary graft nonfunction and retransplantation in 4 patients (2.7%), rejection during ICU in 13 patients (8.8%) after a median of 10 days (range 4 to 20), reoperations during the ICU stay in 29 patients (19.7%) whereas 21 (14.3%) patients had 1 reoperation, 2 (1.4%) patients had 2 reoperations, 3 (2.0%) patients had 3, 2 (1.4%) patients 4 and 1 patient had 10 reoperations. Taken together the 147 transplant recipients underwent 52 reoperations during their ICU stay. One patient (0.7%) underwent percutaneous coronary intervention after the occurrence of acute coronary syndrome (Figure 1). After transplantation, the serum peak levels of bilirubin was 136 ± 116 μmol/l, alkaline phosphatase was 170 ± 136 U/l, ALT was 1401 ± 1436 U/l and AST was 2199 ± 2734 U/l. The causes for readmission are shown in Table 3.

**Table 3:** Readmission causes (n = 29; 19.7%)

Typ	Number
Neurological	2 (1.4%)
Reanimation after cardiac arrest	1 (0.7%)
Respiratory failure	3 (2.1%)
Renal failure	4 (2.8%)
Liver failure	4 (2.8%)
Gastrointestinal bleeding	2 (1.4%)
Other abdominal pathologies	8 (5.6%)
Infection/sepsis	2 (1.4%)
Others	3 (2.1%)

### How was the mortality rate?

**Figure 2:**
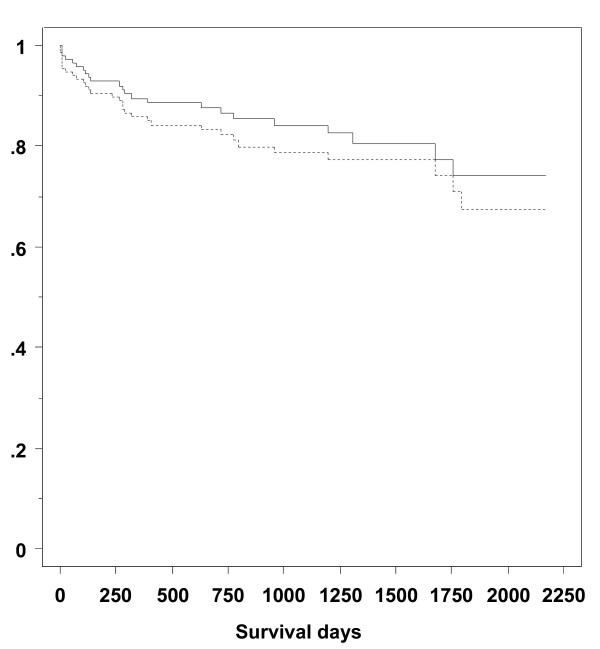
**Kaplan Meier analysis of cumulative graft survival (dashed line) and cumulative patient's survival (full line)**. Graph shows results for 144 patients and 151 grafts.

The ICU mortality was 3.5% (5 of 144 patients) and the hospital mortality was 5.6% (8 of 144 patients). Cumulative graft survival was 86.5% after one year, 79.3% after three years and 67.9% after five years and the cumulative patients survival was 89.5% after one year, 84.1% after three years and 74.1% after five years, respectively (Figure 2).

### Did MELD affect morbidity and mortality?

**Figure 3:**
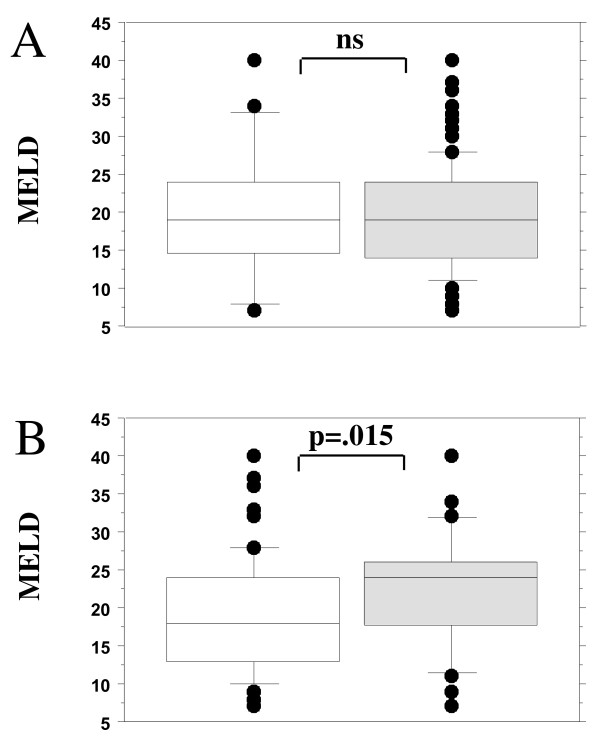
**Influence of MELD score on (a) mortality and (b) length of stay in the ICU of more than 10 days**. There was a significant higher model of end-stage liver disease (MELD) in the group, which stayed longer in the ICU (grey box). In contrast there was no difference in MELD in respect to mortality. **(a) **24 no survivors vs. 104 survivors. **(b) **35 with a long ICU stay versus 93 short time ICU patients. ns, not significant.

MELD score corrected was significantly increased in the patients, which stayed longer than 10 days in the ICU (22.3 ± 7.6 vs. 18.8 ± 7.2, *P *= 0.015), but had no influence on mortality (Figure 3). The odds ratio for longer (> 10 days) ICU stay was 7.0 (confidence interval: 1.7 to 28.4, *P *= 0.007).

### What are the risk factors for mortality?

The Cox proportional hazard model for mortality identified sepsis (*P *= 0.011), postoperative RRT on ICU (*P *= 0.002), transfusion of more than 7 units of RBC (*P *= 0.045) and hepatorenal syndrome before transplantation (*P *= 0.016) as independent risk factors for mortality. Transfusion of more than 10 units of FFP, gender, use of marginal grafts, age, pretransplant diabetes mellitus, or postoperative bilirubin peak level, did not affect mortality (Table 4).

**Table 4:** Cox proportional hazard model for mortality

Parameter	*P *value	Hazard ratio	Confidence interval
Incidence of HRS pre TPL	0.016	13.2	1.6-108.8

Sepsis in ICU	0.011	8.9	1.6-47.6

Transfusion > 7 RBC	0.045	7.6	1.04-55.6

Renal replacement therapy in ICU	0.002	6.8	2.0-22.7

Use of marginal grafts	0.39	1.6	0.6-4.6

Transfusion > 10 FFP	0.93	1.0	0.9-1.1

Peak bilirubin serum level	0.25	1.0	0.9-1.1

Diabetes mellitus preoperative	0.65	1.3	0.4-4.4

MELD > 23	0.26	1.1	0.9-1.3

Gender	0.56	1.4	0.4-4.9

Age	0.41	1.0	0.9-1.1

FFP, fresh frozen plasma; HRS, hepatorenal syndrome; MELD, model of end-stage liver disease; RBC, red blood cells; TPL, transplantation.

### What are the risk factors for morbidity?

The multiple logistic regression analysis of predictive factors for ICU length of stay of more than 10 days identified use of marginal grafts (*P *= 0.022), development or renal failure of more than RIFLE class 2 (*P *= 0.006), transfusion of more than 10 units of FFP (*P *= 0.034), respiratory failure (*P *= 0.009), MELD score corrected above 23 (*P *= 0.007), transfusion of more than 7 units of RBC (*P *= 0.032) and sepsis (*P *= 0.046) as independent risk factors. Age, gender, preoperative incidence of diabetes mellitus, directly pretransplantation ICU admission (transplantation from the ICU), postoperative bilirubin serum peak level were no predictors of length of stay in the ICU (Table 5).

**Table 5:** Multiple logistic regression for ICU length of stay of more than 10 days

Parameter	*P *value	Odds ratio	Confidence interval
Sepsis in ICU	0.046	46.7	1.1-2038.1

Respiratory failure	0.009	18.7	2.1-166.1

Transfusion of > 10 FFP	0.034	11.6	1.2-111.7

Transfusion of > 7 RBC	0.032	7.8	1.2-50.5

Renal failure > RIFLE class 2	0.006	7.9	1.9-34.1

MELD score corrected > 23	0.007	7.0	1.7-28.4

Use of marginal grafts	0.022	5.1	2.3-500.0

Gender	0.54	1.0	0.9-1.1

Age	0.08	1.0	0.9-1.1

Diabetes mellitus preoperative	0.46	1.7	0.4-6.4

Transplantation directly from the ICU	0.63	1.6	0.2-10.5

Peak bilirubin serum level	0.45	1.0	0.9-1.1

FFP, fresh frozen plasma; MELD, model of end-stage liver disease; RBC, red blood cells; RIFLE, risk, injury, failure, loss, end-stage of kidney disease.

## Discussion

Currently allocation of liver organs through the MELD system and the impact on patient outcome is a hot debate. Data on the impact of preoperatively assessed MELD score on the morbidity and mortality of postoperative recipients are only few. This study correlated morbidity, but not mortality with the MELD score in patients after liver transplantation in uni- and multivariate analyses and demonstrated a MELD score above 23 to be an independent risk factor for an ICU stay longer than 10 days (odds ratio 7.0). Siniscalchi and colleagues reported a correlation of MELD score and postoperative complications in 242 liver transplants [20]. Interestingly, the MELD scores in that study were similar to our findings (22.8 vs. 22.3 in our study in the high morbidity group and 17.6 vs. 18.8 in the low morbidity group). Another study associated increased length of stay in the ICU in association with high MELD score above 30 [7], but failed to find a difference in mortality. Only in patients exceeding a MELD score of 36, mortality seems to be predicted by MELD as reported from Saab and colleagues [21]. In our population, four patients showed MELD score above 35, three of them died in the postoperative course. In contrast, a study of 340 transplanted patients showed no difference in early death in respect to the MELD score [8]. Several other publications from the USA have also document that MELD score cannot predict survival after liver transplantation [22-24]. Nevertheless, the question of whether very high MELD scores affect mortality remains elusive. Taken together, despite no clear correlation of MELD score and postoperative mortality, there is strong evidence of MELD influencing postoperative morbidity and in turn cost [25].

Another finding of this study was a high incidence of postoperative renal failure and subsequently need for RRT. Cox proportional hazard model revealed RRT in the ICU as an independent risk factor for mortality. RRT in our population was necessary in 21.8% during ICU stay. Other studies reported an incidence ranging from 3% to 20% [26-28] depending on the severity of preexisting renal conditions. Our study population included seven cases of pretransplant RRT already in need of RRT. This fact probably contributed to a higher incidence of renal failure when compared with those studies.

Apart from higher postoperative costs, renal failure and subsequent need for RRT is associated with increased mortality in ICU patients in general [29] and in particular in liver transplant recipients, varying from 27% to 67% depending on the comorbidities [30-33]. There is strong evidence that even mild renal failure after transplantation might lead to longer hospital stay, more infections and increased overall mortality [33-35].

In our study population, 95 (67.9%) patients presented with renal failure at different stages according to the RIFLE criteria. Planinsic and Lebowitz observed renal failure in more than 80% of cases during the first 48 hours after surgery for liver transplantation. Mortality was extremely high in up to 50% of liver transplant recipients with renal dysfunction at 30 days following surgery and, if hemodialysis was required, it could reach 60% [35]. The etiology of renal failure after liver transplantation is certainly multifactorial. Most reported risk factors are pretransplant renal dysfunction, low serum albumin, dysfunction of the liver graft, bacterial infections and reoperations [36].

Furthermore, the contribution of intraoperative stressors is not to be neglected: hypotension with or without hypovolemia, operation without veno-venous bypass [37,38] and use of nephrotoxic agents as antibiotics or immunosuppressants may further contribute to progressive renal failure.

Interestingly in our study population among the patients in need of postoperative RRT, even patients with preoperative normal kidney function could be found, which underlines the impact of intra- and postoperative stressors on renal failure. The focus of postoperative management should lie on provisions to avoid renal failure and logically lower morbidity and mortality.

Looking at preoperative kidney function in our study population we found an incidence of hepatorenal syndrome of 20% with a hazard ratio of 13, which corresponds to other studies [39-41]. Although liver transplantation can correct hepatorenal syndrome [42], the time frame for recovery of renal function seems to be too long for RRT-free management. Often RRT is needed as a bridging therapy until the kidneys recover. In our study population, if duration of dialysis pretransplant was less than 30 days only 8% of patients still require hemodialysis 8 weeks after transplantation, which is somewhat in contrast to the study by Lo and colleagues, where 25% of the patients with hepatorenal syndrome require long-term RRT after transplantation [43]. Thus, hepatorenal syndrome is not always reversed, in particular when pretransplant RRT is necessary [44] and additional kidney transplantation becomes an option [40]. Hepatorenal syndrome prior to transplantation and RRT postoperatively are strong predictors for mortality in liver transplant recipients and the postoperative renal impairment leads to a prolonged ICU stay for these patients.

Allogeneic FFP and RBC transfusions are associated with well-known adverse effects, reflected by increased incidence in viral and bacterial infections, activation of inflammatory and coagulation pathways, and immunologic reactions [45-47]. In patients after liver transplantation, intraoperative transfusion of packed RBCs are associated with more complications [48,49] and infections [50]. Our multivariate analysis revealed transfusion of more than 7 units of RBCs and transfusion of more than 10 units of FFP as independent risk factors for mortality and prolonged ICU stay. Other reports identified intraoperative transfusion as a risk factor for morbidity and mortality in liver transplant recipients [39,50,51] and Massicotte and colleagues could demonstrate that a restrictive transfusion regime was associated with better outcome in liver transplantation recipients with an average MELD of 18 [49]. Thus, avoiding transfusion of RBC seems to be crucial to reduce postoperative morbidity and mortality.

In our group ICU mortality was 3.5% and the hospital mortality was 5.6%. The hospital mortality is closely related to the hospital length of stay [52]. Our survival data are similar to other transplant programs [6-8] with a cumulative patient survival of 89.5% after one year, 84.1% after three years and 74.1% after five years, even though in our study population 38.4% of marginal donor grafts were transplanted. In our study population, the use of marginal liver grafts was associated with higher ICU length of stay, but did not lead to an increased overall mortality [53,54] and is able to decrease wait list mortality.

Sepsis was also highly associated with prolonged ICU stay and increased mortality confirming the results of other studies [55,56]. It is still a leading cause of death (20 to 50%) in non-cardiac ICUs [57]. In our study population sepsis occurred in 10.8%, that is, it was ranked fourth in the complication list after renal failure, readmissions and reoperations.

The gastrointestinal system might play a key role in the pathogenesis owing to a breakdown of intestinal barrier function. Gurusamy and colleagues concluded from their database review that the use of prebiotics and probiotics might be effectful in the prevention of sepsis [58]. However, our patients received no prebiotics or probiotics, but this might be a beneficial therapeutical option in the future. Most importantly these patients should be management according to the guidelines of the Survival Sepsis Campaign [59,60].

## Conclusions

This study identified MELD score above 23 as an independent risk factor of morbidity represented by ICU stay longer than 10 days but it did not clearly affect mortality. This finding supports the transplantation of patients with high MELD score at the cost of increased postoperative morbidity, in particular when it is seen in the light of reduced waiting list mortality. Furthermore, we identified transfusion of more than seven units of RBCs as an independent risk factor for mortality and for prolonged ICU stay. Postoperative renal failure and transfusion of more than 10 units of FFP are strong predictors of morbidity and postoperative RRT was highly associated with increased mortality, as was hepatorenal syndrome prior to transplantation.

## Key messages

• High MELD scores greater than 23 did not affect mortality in liver transplant recipients.

• Sepsis, postoperative RRT on ICU, transfusion of more than seven units of RBC and hepatorenal syndrome before transplantation were strong predictors for mortality in liver transplant recipients.

• Transplantation of marginal grafts, development or renal failure greater than RIFLE class 2, transfusion of more than 10 units of FFP, respiratory failure, MELD score greater than 23, transfusion of more than seven units of RBC and sepsis are predictors for increased length of stay in the ICU.

## Abbreviations

ALT: alanine aminotransferase; ARDS: acute respiratory distress syndrome; AST: aspartate aminotransferase; ESLD: end-stage liver disease; FFP: fresh frozen plasma; MELD: model of end-stage liver disease; RBC: red blood cells; RRT: renal replacement therapy.

## Competing interests

The authors declare that they have no competing interests.

## Authors' contributions

MB and CEO designed the study. CEO and PD performed the study. RS and JFS collected data. RAS analysed data. PAC and MB wrote the paper.
